# Hypertension May Reduce the Infection Risk but Increase the Severity of COVID-19: Based on the Current Data in China

**DOI:** 10.1155/2021/6594863

**Published:** 2021-12-20

**Authors:** Bo Li, Lu Zeng, Nengjun Sun, Yunhe Zhao, Faming Zhao, Hongjun Bian, Wei Yi, Jing Yang, Bin Li, Guohai Su

**Affiliations:** ^1^Department of Cardiology, Shandong University Zibo Central Hospital, No. 10, South Shanghai Road, Zibo 255000, China; ^2^Department of Cardiology, Zibo Hospital Affiliated to Shandong First Medical University, Zibo, Shandong 255000, China; ^3^Weifang Medical University, No. 288, Shengli East Street, Weifang City, Shandong 261000, China; ^4^Department of Medical Administration, Shandong University Central Hospital of Zibo, No. 54, Gong Qing Tuan Xi Road, Zibo 255036, China; ^5^Department of Infectious Diseases, No. 4, Hospital of Zibo, No. 210, Shan Quan Road, Zibo 255000, China; ^6^Departments of Emergency Medicine, Shandong Provincial Hospital Affiliated to Shandong First Medical University, Jinan 250012, China; ^7^Department of Intelligent Manufacturing Teaching, Engineering Training Center, Shandong University, Jinan 250002, China; ^8^Binzhou Medical University, No. 346, Guanhai Road, Laishan District, Yantai 264003, China; ^9^Department of Cardiology, Central Hospital Affiliated to Shandong First Medical University, Jinan, Shandong,250013, China; ^10^Department of Cardiology, Jinan Central Hospital, Cheeloo College of Medicine, Shandong University, Jinan, Shandong 250013, China

## Abstract

Increasing evidence has shown an unusual relationship between hypertension and COVID-19, which may not be as simple as previously thought. The purpose of our study was to determine the association of hypertension with the onset and development of COVID-19. A meta-analysis was performed to summarize the prevalence of hypertension in COVID-19 patients, as well as the usage of ACEIs/ARBs. Metaregression analyses were used to evaluate the association of hypertension with disease severity and mortality. PubMed and Google Scholar were searched for relevant studies. A total of 42 studies including 14138 patients were enrolled in the study. The proportion of hypertension in COVID-19 patients in China was 17.7% according to the enrolled studies, while it was 6.0% in a study containing 72314 confirmed cases, which are both much lower than in the general population. All of the data from the 11 provinces in China showed the same tendency. The proportions of hypertension were higher in severe/ICU patients and nonsurvivors than in nonsevere/ICU patients and survivors. The metaregression analyses suggested that both disease severity and risk of death were associated with the incidence of hypertension. A total of 27.6% of COVID-19 patients with hypertension received ACEI/ARB therapy. The proportion of deaths in COVID-19 patients with hypertension treated with ACEIs/ARBs was significantly lower than that in nonuse patients treated with ACEIs/ARBs. In conclusion, hypertension may reduce the infection risk of COVID-19 but increase the risk of developing worse clinical outcomes. The use of ACEIs/ARBs may benefit COVID-19 patients with hypertension.

## 1. Introduction

With the publication of several studies concerning the epidemiological and clinical features of coronavirus disease 2019 (COVID-19), a growing amount of evidence is emerging about the relationship between cardiovascular complications and COVID-19. According to early published data in Wuhan, hypertension was associated with a high prevalence and an increased risk of morbidity and mortality.

In Wang's study, 31.2% of the 138 confirmed COVID-19 cases had hypertension [1]. Another study included 140 COVID-19 patients, of whom 42 patients (30%) had hypertension [2]. The prevalence of hypertension in fatal patients was extremely high (42.57%–60%) [3–5]. Thus, patients with hypertension seemed to be more likely to be infected with SARS-CoV-2 and face a greater risk of developing severe conditions [6, 7].

However, emerging evidence shows the reported prevalence of hypertension is gradually decreasing. In our previous study, the 6 earliest studies about COVID-19 were summarized, and the results suggested that the proportion of hypertension in patients with COVID-19 was 17.1% [8]. A study that included 1099 patients with confirmed COVID-19 reported that 14.9% of patients had hypertension comorbidities [9]. In another study, among 44672 individuals with confirmed COVID-19, 2683 patients (12.8%) had hypertension [10]. Even more surprising was that in the largest study ever conducted in China thus far, including 72314 confirmed cases, only 6.0% of patients had hypertension [11]. Because the total number of infections in China was 84414 as of May 7th, the study was very representative of the total population. The incidence of 6.0% was much lower than that of the general population, which reported a 27.9% overall crude prevalence of hypertension according to the Summary of the 2018 report on cardiovascular diseases in China [12]. This information indicated that individuals with underlying hypertension seemed to be less susceptible to COVID-19. This discovery is very different from all others that have been made thus far. Thus, it is urgent to determine the relationship between hypertension and the virus.

Angiotensin-converting enzyme inhibitors (ACEIs) and angiotensin receptor blockers (ARBs) are the main antihypertensive drugs recommended in the current guidelines. However, angiotensin-converting enzyme 2 (ACE2) has been identified as a receptor for SARS-CoV-2, which infects humans by binding to ACE2 in host respiratory epithelial cells [13]. Therefore, the application of ACEIs/ARBs to control blood pressure in patients with COVID-19 complicated with hypertension has been a matter of intense debate [14, 15]. Therefore, we will conduct an analysis on the incidence and prognosis of patients with COVID-19 complicated with hypertension, as well as the use of ACEIs/ARBs in infected patients and their impact on prognosis.

## 2. Methods

This meta-analysis was conducted following the Preferred Reporting Items for the Systematic Review and Meta-Analysis (PRISMA) statement [16].

### 2.1. Search Strategy and Selection Criteria

A literature search was conducted in MEDLINE via the PubMed database, Cochrane Library, Web of Science, EMBASE and EMBASE Classic databases, and Google Scholar from inception through April 23, 2020. The following terms (MeSH) were used: “2019-nCoV,” “COVID19 virus,” “2019 novel coronavirus,” “SARS-CoV-2,” and “hypertension.” The included articles met the following eligibility criteria: (1) RCTs or non-RCTs; (2) patients were diagnosed with 2019 novel coronavirus infection; (3) sample size ≥ 10 in each study population; and (4) comorbidities of hypertension and the outcome of hypertension were given. Studies not meeting these criteria, nonclinical studies, incompatible or repeated studies, case reports, and studies without complete data were excluded.

### 2.2. Data Management and Quality Assessment

Clinical data, including age, sex, prevalence of hypertension, clinical outcome, and interventions for hypertension, were collected from the identified studies. The primary outcomes were to analyse the prevalence of hypertension and the impact of hypertension on the severity of the disease and mortality. The secondary outcomes were to analyse the use of ACEIs/ARBs in COVID-19 and the impact of ACEIs/ARBs on prognosis. Two reviewers assessed the eligibility of the included reports, extracted data with a standardized report form, and evaluated the quality of reports independently. All discrepancies were solved by discussion until a consensus was reached.

### 2.3. Statistical Analysis

The statistical analysis was performed using OpenMeta Analyst version 10.10 (https://www.cebm.brown.edu/open_meta) and Review Manager (RevMan) software version 5.3. Forest plots were used to illustrate the prevalence of hypertension in 2019-nCoV infection from the selected studies as well as the impact of 2019-nCoV infection and therapeutic interventions on hypertension. Heterogeneity among studies was assessed by *I*^2^ statistics. In the pooled analysis, a fixed-effects model was applied when the heterogeneity *I*^2^ was less than 50%; otherwise, a random-effects model was used [17, 18]. Metaregression analyses were used to evaluate the associations between disease severity, risk of death, and incidence of hypertension. Cochrane Collaboration's tool was followed to assess the risk of bias.

## 3. Results

### 3.1. Selected Studies and Baseline Characteristics

**Figure 1:**
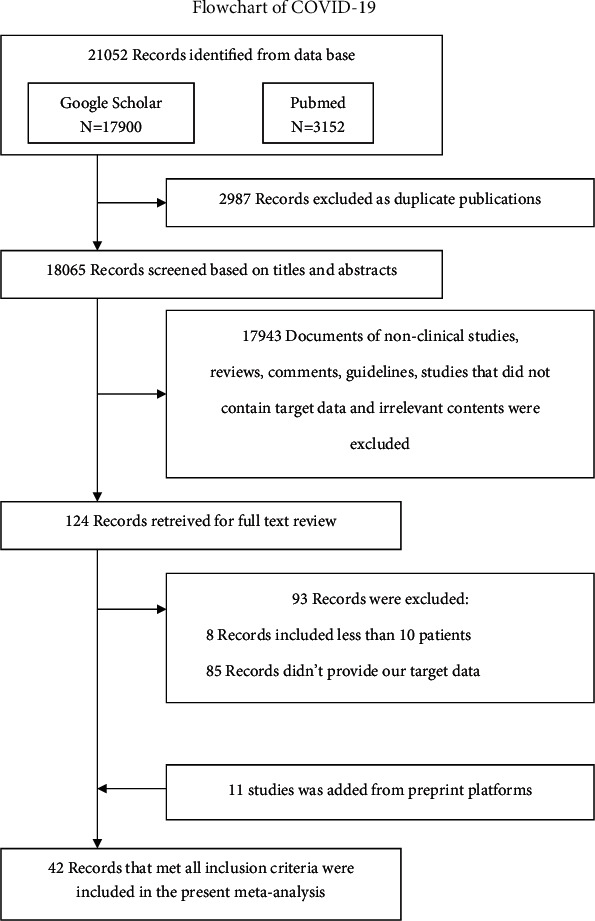
Flow diagram of the study selection process.

We searched all the databases and websites using the search words described above, and 21052 total records were retrieved. Among the articles, 2987 records were excluded because of duplication. In addition, 17943 records were excluded because they were animal trials, letters, review articles, case reports, or repetitive content. The remaining 124 studies were carefully evaluated in detail. Finally, 93 studies were excluded because the participants of the trials did not meet the criteria we set. Then, 11 studies published in the preprint platform were added. Finally, a total of 42 articles were selected for this study (Table 1 and Figure 1).

### 3.2. Primary Outcomes

**Figure 2:**
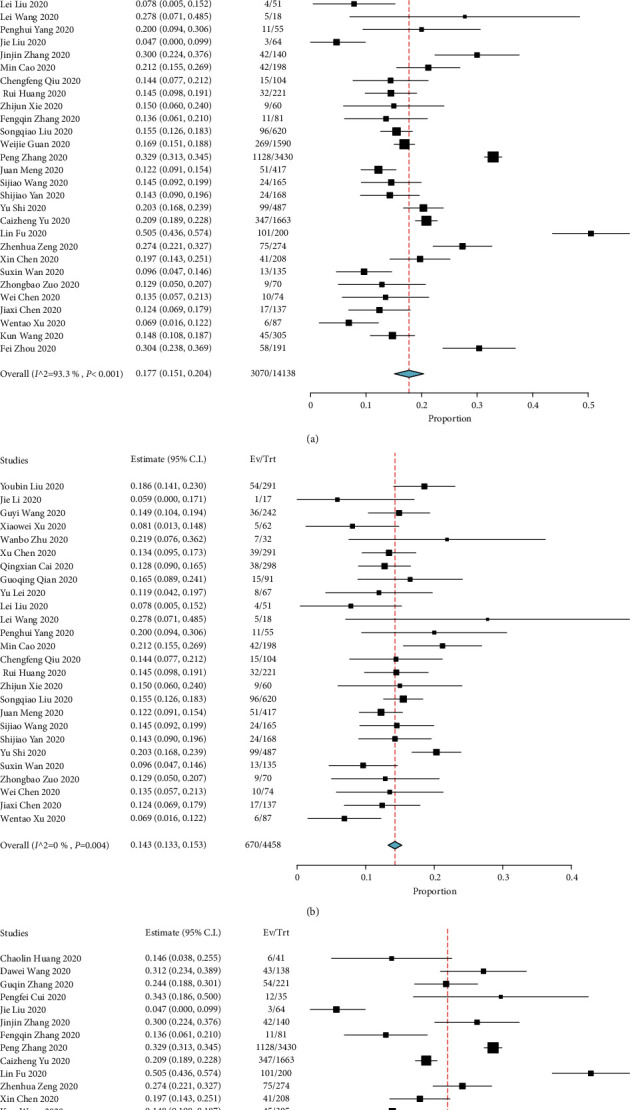
Meta-analysis for the proportion of hypertension in COVID-19 cases. Weights were calculated from binary random-effects model analysis. Values represent the proportion of hypertension in COVID-19 patients and 95% CI. Heterogeneity analysis was carried out using the Q test among the studies variation (*I*^2^ index). (a) The proportion of hypertension in data from all of China. (b) The proportion of hypertension outside Hubei. (c) The proportion of hypertension in Hubei.

Forty-two studies, including 14138 COVID-19 patients with hypertension, were collected in the study. Meta-analysis of the identified studies showed that the prevalence of hypertension was 17.7% (95% CI: 15.1–20.4%) (Figure 2(a)). There was significant heterogeneity (Cochran's Q) in the estimates of hypertension among the identified studies with an *I*^2^ index of 93.3%. Next, 26 studies including 4458 patients outside of Hubei were also analysed, showing that the prevalence of hypertension was 14.3% (95% CI: 13.3–15.3%) (Figure 2(b)). Then, 14 studies including 6991 patients in Hubei Province were analysed, and the data showed that the prevalence of hypertension was 24.7% (95% CI: 19.4–30.1%) (Figure 2(c)).

**Figure 3:**
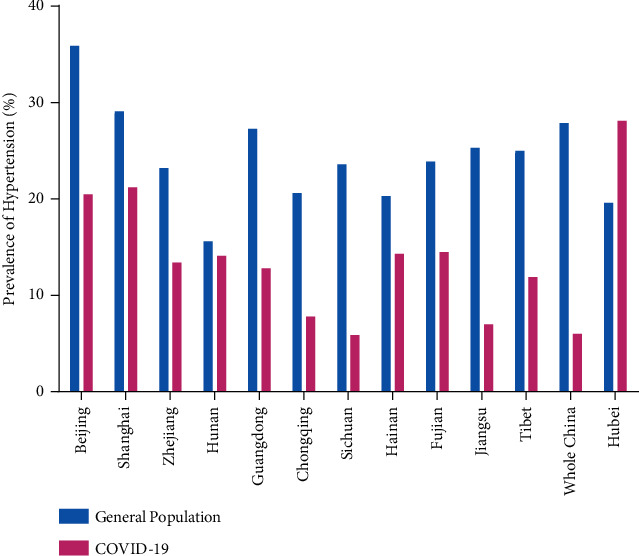
Comparison of the incidence rates of hypertension in COVID-19 patients with that from the China Hypertension Survey, 2012–2015, in 11 provinces and all of China.

Furthermore, we compared the prevalence of hypertension in COVID-19 patients with data from the China Hypertension Survey, 2012–2015 [19]. The results demonstrated the differences in all 11 provinces outside of Hubei whose data could be currently obtained (Figure 3) as follows: Beijing (35.9% vs. 20.5%) [20, 21], Shanghai (29.1% vs. 21.2%) [22], Zhejiang (23.2% vs. 13.4%) [23–25], Hunan (15.6% vs. 14.1%) [26–28], Guangdong (27.3% vs. 12.8%) [29], Chongqing (20.6% vs. 7.8%) [30], Sichuan (23.6% vs. 5.88%) [31], Hainan (20.3% vs. 14.3%) [32], Fujian (23.9% vs. 14.5%) [33], Jiangsu (25.3% vs. 7.0%) [34], and Tibet (25.0% vs. 11.9%) [35] (Figure 3).

**Figure 4:**
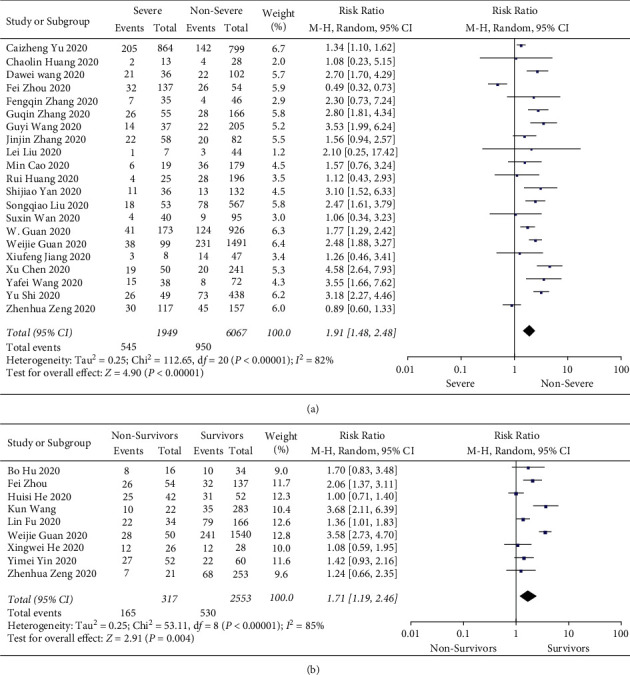
(a) Forest plots depict the comparison of the incidences of hypertension in severe/ICU and nonsevere/ICU patients. (b) Forest plots depict the comparison of the incidences of hypertension in nonsurvivors and survivors. Forest plots depict the comparison of the incidences of cardiac injury in ICU/severe and non-ICU/severe patients.

**Figure 5:**
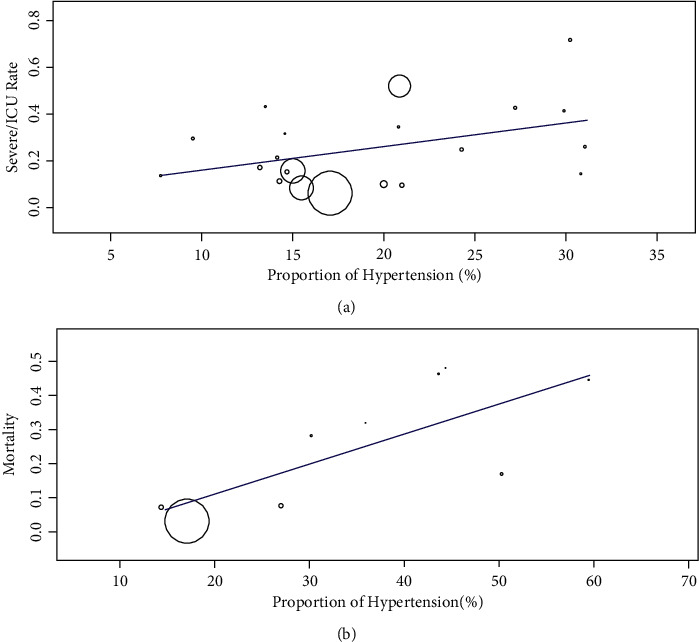
(a) Bubble plots for the association of hypertension with severe/ICU rates in COVID-19 cases. (b) Bubble plots for the association of hypertension with mortality in COVID-19 cases.

Then, the differences in the prevalence of hypertension between severe/intensive care unit (ICU) and nonsevere/ICU patients and nonsurvivors and survivors were compared. There was significant statistical heterogeneity in the test results (severe/ICU and nonsevere/ICU patients: *I*^2^ = 82%; nonsurvivors and survivors: *I*^2^ = 85%). Therefore, a random-effects model was selected for further analysis. Studies with a total of 8004 patients showed that hypertension accounted for 28.0% of severe/ICU cases, but only 15.7% of nonsevere/ICU cases. The proportion of hypertension was significantly higher in severe/ICU patients than in nonsevere/ICU patients (RR = 1.91, 95% CI (1.48, 2.48), *Z* = 4.90, *P* < 0.00001) (Figure 4(a)). For nonsurvivors and survivors with hypertension (with a total number of 2870 patients), the heterogeneity test showed that *I*^2^ = 85%, and a random effects model was used. Hypertension was present in 52.1% of nonsurvivors but 20.8% of survivors. The results indicated a much higher proportion of hypertension in nonsurvivors than in survivors, with statistical significance (RR = 1.71, 95% CI (1.19, 2.46), *Z* = 2.91, *P*=0.004) (Figure 4(b)). In metaregression analyses of the included studies, disease severity and risk of death were both associated with the incidence of hypertension (*β* = 0.010, *P*=0.032 and *β* = 0.009, *P* < 0.001, respectively) (Figures 5(a) and 5(b)).

### 3.3. Secondary Outcomes

**Figure 6:**
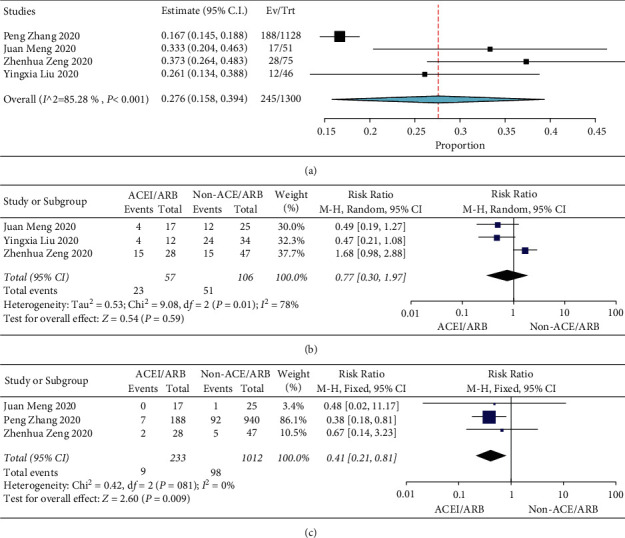
(a) Prevalence of usage of ACEIs/ARBs in COVID-19 patients with hypertension. (b) Comparison of mortality in ACEI/ARB and non-ACEI/ARB patients. (c) Comparison of the incidences of the severe/ICU rate in ACEI/ARB and non-ACEI/ARB patients.

Finally, we collected data from COVID-19 patients with hypertension according to the use of ACEIs/ARBs. Four studies (including 1300 patients) showed that 27.6% (95% CI: 15.8–39.4%) of COVID-19 patients with hypertension received ACEI/ARB therapy (Figure 6(a)). Then, we compared the differences in the severe/ICU rate and mortality of COVID-19 patients with hypertension between the ACEI/ARB group and the non-ACEI/ARB group. Meta-analysis showed that the proportion of deaths in COVID-19 patients with hypertension treated with ACEIs/ARBs was significantly lower than that in nonuse patients treated with ACEIs/ARBs (RR = 0.41, 95% CI (0.21, 0.81), *Z* = 2.60, *P*=0.009) (Figure 6(b)). The results also showed a slightly lower proportion of severe/ICU among patients treated with ACEIs/ARBs than in patients not treated with ACEIs/ARBs, but the difference was not statistically significant (RR = 0.77, 95% CI (0.30, 1.97), *Z* = 0.54, *P*=0.59) (Figure 6(c)).

## 4. Discussion

A growing number of studies on the epidemiological and clinical characteristics of COVID-19 suggest a relationship between cardiovascular complications and COVID-19. Hypertension is one of the most important complications in patients with COVID-19. According to earlier data in Wuhan, there was a high proportion of hypertension among confirmed cases, reaching more than 30%. However, in a study covering 72314 confirmed cases, only 6.0% of patients had hypertension [11]. In the present study, we collected all of the published data involving provinces outside Hubei. We compared the prevalence of hypertension in COVID-19 patients with the data from the China Hypertension Survey, 2012–2015 [19]. The results demonstrated the differences in all 11 provinces whose data could be currently obtained. All of the incidences of hypertension in COVID-19 patients were lower than those in the general population. We do not believe this is just a coincidence. We hypothesized that hypertension might reduce the risk of SARS-CoV-2 infection.

However, in Hubei Province, the result was the opposite. One possible reason why the incidence of hypertension in COVID-19 patients was higher than that of the general population in Hubei was that many people with mild illnesses might have gone undiagnosed in the face of a collapsed health care system, which would reduce the accuracy of the data. However, when the healthcare system is healthy, milder and even asymptomatic patients can be diagnosed, which might result in a lower prevalence of hypertension outside of Hubei.

If the above hypothesis and interpretation can be accepted, there must be a premise as follows: the incidence of hypertension in mild patients is low, but it is high in severe patients. To test this premise, we analysed the prevalence of hypertension between severe and nonsevere patients and nonsurvivors and survivors. We found that patients with hypertension were more likely to develop severe cases or be nonsurvivors after SARS-CoV-2 infection. The total proportion of hypertension in severe/ICU patients was approximately 1.78-fold that in nonsevere/ICU patients, while in nonsurvivors, it was 2.50-fold that in survivors, both of which showed significant differences. In metaregression analyses in patients with COVID-19, the disease severity and risk of death were both statistically associated with the incidence of hypertension.

Although the underlying mechanism is unknown, further investigation of the expression and activity of ACE2 is worthwhile. ACE2 is a carboxypeptidase that can hydrolyse Ang I to Ang-(1–9) and Ang II to Ang-(1–7) without being inhibited by selective ACE inhibitors. ACE2 has more favourable kinetics for the hydrolysis of Ang II than Ang I. [36] A previous study suggested that the ACE2 expression level was significantly downregulated in the kidneys of three hypertensive rat strains [37]. In a clinical study, hypertension status was also confirmed as an independent confounding determinant of the ACE to ACE2 ratio, leading to the relative downregulation of ACE2 [38]. SARS-CoV-2 binds to its target cells through ACE2, which is expressed in epithelial cells, type 2 pneumocytes, and macrophages in the lungs. Studies have speculated that high expression of ACE2 in patients with hypertension might facilitate SARS-CoV-2 entry into targeted cells in the respiratory system [13]. One of the main reasons why hypertension could reduce SARS-CoV-2 infection might be the loss of ACE2 in hypertensive subjects. ACE2 has a strong cardiovascular protective effect, which could also explain why patients with hypertension had a worse prognosis once they were infected with the virus. The downregulated expression of ACE2 after infection with SARS-CoV leads to excessive activation of the RAS [39], which activates peripheral sympathetic neuron release of catecholamine mediated by activation of multiple signalling pathways causing vasoconstriction and bronchial contraction [40]. It has been shown that the binding of coronavirus to ACE2 leads to the downregulation of ACE2, which in turn causes an ACE/ACE2 imbalance and an excessive production of angiotensin II by the related ACE enzyme. Excessive activation of the RAS also promotes inflammation, causes cytokine storms [41], generates ROS by activating the NADH/NADPH oxidase system [42], and induces cell apoptosis, thus promoting the progression of coronavirus-related lung injury [43].

ACEIs and ARBs are the major RAS inhibitors commonly used in clinical practice and the main antihypertensive drugs recommended in current guidelines. Although ACE is not directly inhibited by ACE inhibitors, ACE2 is affected by chronic treatment with these drugs, which leads to an increase in ACE2 expression in several tissues [44]. Interestingly, this feature is also shared by another drug, angiotensin receptor-1 blocker (ARB). According to the circulating level of the ACE2 product angiotensin 1–7, long-term administration can also increase the expression level and activity of ACE2 [45]. The use of ACEIs/ARBs in COVID-19 patients with hypertension has caused great controversy. We performed a meta-analysis on the application of ACEIs/ARBs in patients with COVID-19 and hypertension and found that 27.6% (95% CI: 15.8–39.4%) of patients received ACEI/ARB therapy. The proportion of nonsurvivors or severe patients with hypertension and COVID-19 treated with ACEIs/ARBs was lower than that of survivors or nonsevere patients. The use of ACEIs/ARBs upregulated the expression of ACE2, which might increase susceptibility to COVID-19 and in turn reduce the severity of the disease. In Zhang's study, among hospitalized COVID-19 patients with hypertension, the use of ACEIs/ARBs was associated with a lower risk of all-cause mortality. The upregulation of ACE2 induced by long-term intake of AT1R and ACE inhibitors may play a protective role through the following two mechanisms, first, by blocking the increase of angiotensin 1–7 and, second, by reducing the production of angiotensin II [46].

There were several limitations in the present study. First, there was a difference in the proportion of hypertension among the included cohorts, which may account for the heterogeneity. Occasionally, a small number of samples were reused, which might lead to bias; however, they cannot be excluded crudely in order to avoid bias caused by incomplete data inclusion. Second, the differences in sample size and study design in different studies may be one of the reasons for the heterogeneity. Third, in different studies, severe patients are defined according to different criteria. To simplify the conclusions and improve readability, we combined the severe patients and ICU patients into one category for analysis. In addition, confounding factors (such as sex, smoking, drinking, and history of other comorbidities) in studies may have impacted the outcomes of the patients.

## Figures and Tables

**Table 1:** Number, age, sex, and hypertension of patients of the included studies.

References	Date	Number of patients	Area	Age	Sex (male, %)	Hypertension (%)
Chaolin Huang	As of 2020.1.2	41	Wuhan	49	73.2	14.6
Youbin Liu	2020.1.10–2020.2.24	291	Guangzhou	48.1	45.7	18.6
Jie Li	2020.1.22–2020.2.10	17	Dazhou	45	52.9	5.9
Guyi Wang	As of 2020.2.20	242	Changsha	45	49.2	14.9
Yafei Wang	2020.1.1–2020.2.10	110	Wuhan	—	43.6	20.9
Xiaowei Xu	2020.1.10–2020.1.26	62	7 hospitals in Zhejiang	41	56.5	8.1
Wanbo Zhu	2020.1.24–2020.2.20	32	Hefei	46	46.9	21.9
Xu Chen	2020.1.23–2020.2.14	291	Changsha and Loudi	46	49.8	13.4
Qingxian Cai	2020.1.11–2020.2.6	298	Shenzhen	47	50	12.8
Dawei Wang	2020.1.1–2020.1.28	138	Wuhan	56	54.3	31.2
W. Guan	2019.12.11–2020.1.29	1099	552 hospitals	47	58.1	15.0
Fei Zhou	2019.12.29–2020.1.31	191	Wuhan	56	62.3	30.4
Guoqing Qian	2020.1.20–2020.2.11	91	7 hospitals in Zhejiang	50	40.7	16.5
Guqin Zhang	2020.1.2–2020.2.10	221	Wuhan	55	48.9	24.4
Pengfei Cui	2020.1.28–2020.2.18	35	Wuhan	61.5	0	34.3
Yu Lei	2020.1.4–2020.2.28	67	Daofu	39.3	58.2	11.9
Lei Liu	2020.1.20–2020.2.3	51	Chongqing	45	62.7	7.8
Lei Wang	2020.1.21–2020.2.5	18	Zhengzhou	39	55.6	27.8
Penghui Yang	2019.12.27–2020.2.18	55	Beijing	44	60	20
Jinjin Zhang	2020.1.16–2020.2.3	140	Wuhan	57	50.7	30
Jie Liu	2020.1.16–2020.2.15	64	Wuhan	35	35.9	4.7
Min Cao	2020.1.20–2020.2.15	198	Shanghai	50.1	51.0	21.2
Chengfeng Qiu	2020.1.22–2020.2.12	104	Huaihua	43	47.1	14.4
Rui Huang	2020.1.22–2020.2.10	221	10 hospitals in Jiangsu	45	57.0	14.5
Zhijun Xie	2020.1.22–2020.2.15	60	Hangzhou	45	45	15
Fengqin Zhang	2020.1.30–2020.2.15	81	Jinzhou	—	55.6	13.6
Songqiao Liu	2020.1.10–2020.2.18	620	24 hospitals in Jiangsu	44.48	52.6	15.5
Weijie Guan	2019.12.11–2020.1.31	1590	575 hospitals	48.9	57.3	17.0
Peng Zhang	2019.12.31–2020.2.20	3430	9 hospitals in Hubei	—	32.9	32.9
Juan Meng	2020.1.11–2020.2.23	417	Shenzhen	64.5	—	12.2
Sijiao Wang	2020.1.22–2020.2.16	165	Fuzhou	44	55.8	14.5
Shijiao Yan	2020.1.22–2020.3.13	168	Haikou	51	48.2	14.3
Xiufeng Jiang	2020.1.23–2020.2.16	55	Wuxi	45	49.1	30.9
Yu Shi	2020.1.16–2020.2.17	487	5 hospitals in Zhejiang	46	53.2	20.3
Huisi He	2020.2.8–2020.3.16	94	Wuhan	69.2	57.4	59.6
Caizheng Yu	2020.1.14–2020.2.28	1663	Wuhan	64	50.4	20.9
Lin Fu	2020.1.1–2020.1.30	200	Wuhan	—	49.5	50.5
Zhenhua Zeng	2020.1.5–2020.3.8	274	Wuhan	60	54.7	27.4
Xin Chen	2020.2.11–2020.2.29	208	Xiaogan	50.5	51.4	19.7
Xingwei He	2020.2.3–2020.2.24	54	Wuhan	68	63.0	44.4
Bo Hu	2020.1.8–2020.2.9	50	Wuhan	62	68	36
Suxin Wan	2020.1.23–2020.2.8	135	Chongqing	47	53.3	9.6
Yimei Yin	As of 2020.2.15	112	Wuhan	66	68.8	43.8
Zhongbao Zuo	2020.1.20–2020.2.28	70	Hangzhou	43	41.4	12.9
Wei Chen	2020.1.19–2020.2.7	74	Nanjing	48.1	58.1	13.5
Jiaxi Chen	2020.1.22–2020.2.26	137	Taizhou	—	52.6	12.4
Wentao Xu	2020.1.10–2020.2.18	87	Suzhou	—	52.9	6.9
Yingxia Liu	2020.1.11–2020.2.5;	78	Shenzhen	—	—	100
	2020.1.12–2020.2.9;		Wuhan			
	2019.12.27–2020.2.27		Beijing			
Kun Wang	2020.1.7–2020.2.11	305	Wuhan	47.8	46.6	14.8
Fei Zhou	2019.12.29–2020.1.31	191	Wuhan	56	62.3	30.4

## Data Availability

The data supporting the findings of this study are included within the article.

## Additional Points

Notwithstanding these limitations, our pooled analysis showed that patients with hypertension might be less susceptible to SARS-CoV-2. However, patients with previous hypertension may be at increased risk of developing worse clinical outcomes and even death. ACEIs/ARBs can control the blood pressure of COVID-19 patients with hypertension, which may improve the prognosis and reduce mortality.
